# Nozzle Angle Optimization for Nasopharyngeal Drug Delivery: In Vitro Penetration Testing and Human Factors Usability Validation

**DOI:** 10.7759/cureus.111659

**Published:** 2026-06-28

**Authors:** César Alas-Pineda, Mohammad Mehedi Hasan Akash, Abir Malakar, Carlos Coto-Tejeda, Jhacely Medina-Mejía, Dennis J Pavón-Varela, Kristhel Gaitán-Zambrano, Gustavo Ferrer, Saikat Basu

**Affiliations:** 1 Department of Research and Development, Moxie Health Group, Hallandale Beach, USA; 2 Department of Mechanical Engineering, South Dakota State University, Brookings, USA; 3 Department of Mechanical Engineering, Florida State University, Tallahassee, USA; 4 Department of Pulmonary and Critical Care Medicine, Aventura Hospital and Medical Center, Aventura, USA

**Keywords:** drug delivery system, feasibility study, intranasal drug administration, nasopharyngeal drug deposition, spray penetration

## Abstract

Background: Intranasal delivery offers a noninvasive route of administration. This proof-of-concept and feasibility study evaluates whether nozzle angle is a key factor in posterior nasopharyngeal deposition, given the limited research on this parameter.

Methods: In Part I (in vitro), nasopharyngeal outflow was quantified in a CT-derived, 3D-printed anatomic nasal cast using a pharma-grade pump sprayer at 12-15° and 67.5° nozzle orientations (5-mm insertion depth; n = 5 replicates/angle/nostril; N = 20 trials); orientations were compared using Mann-Whitney U tests (α = 0.05, two-sided). In Part II (human factors), a nine-step application protocol was evaluated among 191 lay users (simulated use, n = 50; real use, n = 141); stepwise task completion rates with 95% confidence intervals were assessed against a prespecified ≥80% acceptance threshold for essential steps.

Results: The 12-15° orientation yielded higher mean nasopharyngeal penetration per actuation than the 67.5° orientation in both the right nostril, 0.0649 vs. 0.0296 mL (difference, 0.0353 mL; p < 0.001), and the left nostril, 0.0581 vs. 0.0505 mL (difference, 0.0076 mL; p = 0.019). The in vitro section of this study was conducted in a single CT-based anatomical model. In the HF study, all essential task completion rates exceeded 80% in both cohorts, with global mean completion rates of 90.8 ± 8.2% (simulation) and 86.1 ± 11.1% (real use).

Conclusions: A 12-15° nozzle angle produced greater nasopharyngeal penetration than the conventional orientation in a single-anatomy cast model. Lay users followed the optimized protocol with acceptable fidelity for essential steps. Validation across diverse anatomical nasal models and clinical settings is needed before translation into clinical practice.

## Introduction

Intranasal drug delivery exploits the high vascularity and permeability of the nasal mucosa to achieve rapid systemic absorption while bypassing hepatic first-pass metabolism [1,2]. The route is noninvasive, permits self-administration, and has expanded beyond local therapy to include nose-to-brain transport, vaccine delivery, and systemic administration of peptides and nucleic acids [1,3-6]. Yet the clinical performance of any intranasal formulation depends not only on its pharmacology but also on where the spray plume deposits within the nasal cavity [7,8].

Standard over-the-counter nasal spray instructions typically direct users to insert the nozzle nearly vertically and aim toward the ipsilateral eye, producing a spray axis at approximately 60-70° from the horizontal [9]. This orientation favors deposition on the anterior turbinate and largely bypasses the nasopharynx [7,10]. The nasopharynx, however, is the primary site of early viral replication in respiratory infections such as SARS-CoV-2 (severe acute respiratory syndrome coronavirus 2), as confirmed by nasopharyngeal samples [11]. However, the effectiveness of nasal drug delivery may be limited by anatomical and physiological factors, such as mucociliary clearance, complex airflow dynamics, and resistance at the nasal valve [12]. Particular attention needs to be paid to optimizing spray technique and device design to address limitations in drug administration and improve therapeutic efficacy.

Recent computational fluid dynamics (CFD) studies have identified that nozzle insertion depth and spray axis angle are the dominant determinants of posterior deposition, with angles near 10-15° from horizontal producing the highest predicted nasopharyngeal coverage [7,8,10,13,14]. These computational models provide a comprehensive analysis of inhaled airflow and intranasally sprayed particle transport, demonstrating the close relationship between device design and deposition along the airway walls. They facilitate a detailed examination of flow parameters and forces [15]. Vishnumurthy et al. report that particle size and the impaction parameter are the most significant CFD predictors of drug deposition, while inhalation and a tilted head should be further studied. This statement highlights the need for standardized CFD protocols to improve clinical translation of the findings [16]. These computational predictions require experimental validation in anatomic models and critical evaluation to demonstrate that users can execute the correct technique in real-world settings. It is crucial that users follow every step consistently, as variations in technique can affect device performance.

This study addresses both gaps through a two-part proof-of-concept and feasibility design. We tested two explicit primary hypotheses: Part I: an angle of 12-15° produces greater nasopharyngeal penetration compared to the conventional orientation (67.5° from horizontal). Part II: lay users, when provided specific written and visual instructions, can follow a nine-step application protocol with fidelity, achieving ≥80% completion of crucial steps.

Part I presents in vitro spray penetration tests in a 3D-printed, CT-derived anatomic nasal cast comparing nasopharyngeal outflow volumes at 12-15° versus 67.5° nozzle orientations. Part II reports a human factors (HF) usability study in which 191 lay users performed the optimized administration protocol under two conditions: 50 participants applied the spray to a 3D-printed mannequin head under simulated conditions, and 141 participants self-administered the spray following written and visual instructions provided previously. Together, these data provide an initial assessment of both the physical feasibility and the real-world practicality of angle-optimized intranasal delivery.

Importantly, this is a proof-of-concept study; it was not designed to define clinical or therapeutic efficacy. These validations require future studies focused on pharmacokinetic and phase 2 and 3 clinical trials. This study provides a physical and practical feasibility assessment of angle-optimized intranasal delivery.

## Materials and methods

Study design

This proof-of-concept and feasibility study comprised two components conducted sequentially. Part I consisted of in vitro bench-top spray tests to quantify nasopharyngeal penetration inside a representative 3D-printed anatomical upper airway cast for two nozzle orientations. Part II consisted of an observational HF usability study evaluating lay-user adherence to a nine-step application protocol for the optimized orientation. The two components share the same nasal pump sprayer device but address distinct research questions (physical performance vs. user interaction) and are analyzed independently, as neither was designed to establish clinical efficacy or generalizability.

Study objectives

This proof-of-concept and feasibility study had two primary objectives: (1) Part I: to test the hypothesis that a low-angle nozzle orientation achieves greater nasopharyngeal penetration. We hypothesized that this angle would improve posterior nasopharyngeal outflow by at least 25% per actuation. (2) Part II: To evaluate the feasibility of lay users following a nine-step protocol. According to human factors validation guidance, we expect a task-completion rate of ≥80% for the crucial steps.

Participants

We recruited targeted users with the following criteria: lay-user status, both sexes, age range 35-71 years, right- or left-handedness, and with or without experience using an intranasal spray.

The objective of this exploratory investigation was to detect differences in posterior nasopharyngeal penetration. A sample of n = 5 replicates per deposition angle provides 80% power to detect these differences at an α = 0.05 using a two-sided Mann-Whitney U test.

Part I: In vitro spray penetration tests

Anatomic Cast Model

**Figure 1:**
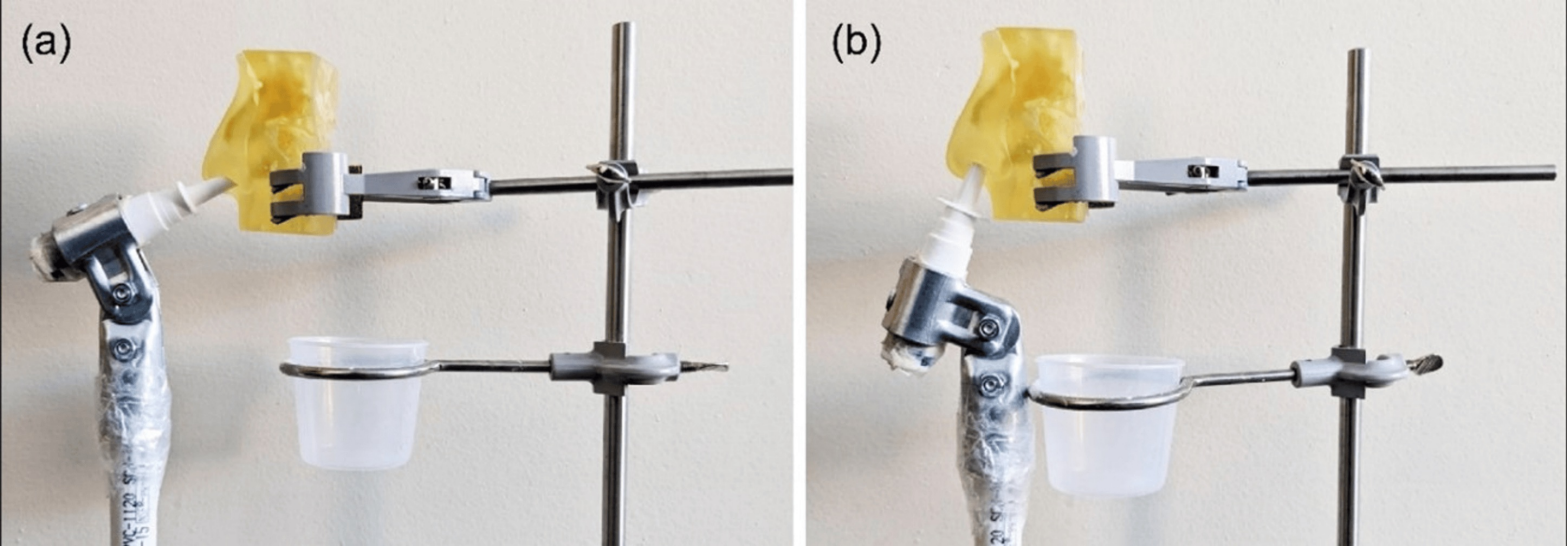
Experimental setup for spray administration into the anatomic 3D-printed nasal cast (a) Nozzle positioned at 12-15° from horizontal. (b) Nozzle positioned at 67.5° from the horizontal, as per standard over-the-counter instructions.

A 3D-printed nasal cavity cast was fabricated from high-resolution, medical-grade computed tomography (CT) imaging data of a 41-year-old Caucasian male. The cast reproduced the anterior nasal passage through a plane immediately anterior to the nasopharynx, allowing collection of posterior outflow as a surrogate for nasopharyngeal deposition. The source CT imaging was acquired on a clinical scanner with a slice thickness of 0.352 mm. The 3D printing was performed with a 0.1 mm layer using Tango Gray FLX950 material (Figure 1). The decision to use a cast from a single donor was intentional to maintain control of all variables except the nozzle angle. The septal deviation identified is addressed in the Discussion; this highlights the need for studies involving diverse anatomy. 

Spray device and test fluid. All tests were conducted using a pharmaceutical-grade nasal pump sprayer. The test fluid was a saline-based solution with a density of 1.3 g/mL. Although physicochemical characteristics were not experimentally determined for this study, the manufacturer's specifications indicate that viscosity and surface tension are within the ranges normally reported for nasal spray formulations (1.28 cP and 56 dynes/cm, respectively) [17,18]. The device was fixed at the target angle using a 0.75-inch adjustable-angle hinge connector, and the cast was secured with a standard burette clamp (Figure 1). The nasal pump sprayer consisted of a high-density polyethylene bottle containing 15 mL of solution, a dust cap, an actuator, a pump, a PE gasket, and a dip tube. The spray pattern showed a circular area ratio of 1.0, with a mean droplet diameter of around 1.2 microns.

Standardization Procedures

The nasal pump sprayer was subjected to strict calibration and consistency through several procedures. The spray force was tested across 10 pre-experimental actuations to ensure force consistency. Between trials, the nasal sprayer was refilled with the saline-based solution, placed in the designated location, and allowed to equilibrate for 40 seconds before the next trial. All trials were performed in a climate-controlled setting (71-75°F) to maintain the solution's integrity.

Protocols

**Figure 2:**
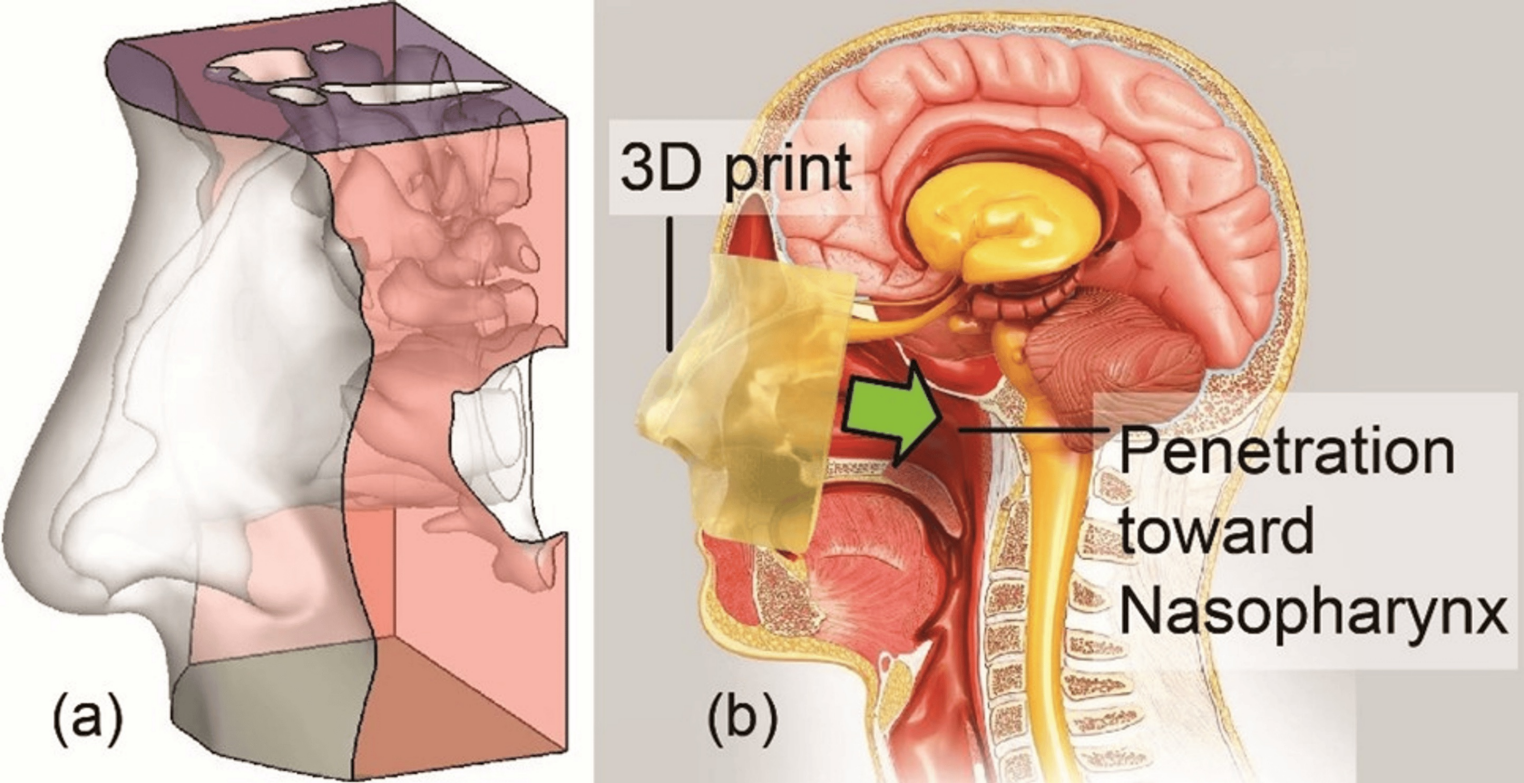
3D-printed anatomic cast (a) Perspective view of the CT-based reconstruction used to fabricate the 3D-printed anatomic cast. (b) Virtual placement of the cast against a sagittal upper airway diagram illustrating the anatomic relationship to the nasopharynx. Figure 2B was partially adapted from Figure 9 of Akash et al. [8] under Creative Commons Attribution (CC BY) license.

Two nozzle orientations were tested: (a) 12-15° from horizontal with a 5-mm insertion depth, aligned with prior CFD-optimized recommendations [7,8,13]; and (b) 67.5° from horizontal with a 5-mm insertion depth, replicating standard over-the-counter nasal spray instructions (Figure 2) [9]. For each orientation, the device was actuated into the right and left nostrils separately until a cumulative posterior outflow target volume of 5.0 mL was collected from the back of the cast; any anterior discharge was collected in a separate container and excluded. Five replicate trials were performed at each angle and nostril (N = 20 trials total). The mean posterior outflow volume per actuation was computed as 5.0 mL divided by the number of actuations per trial. The experimental setup permitted a perturbation of approximately ±1.5° on the 12-15° alignment to account for realistic usage variability. 

Part II: Human factors usability study

Participants

A total of 191 lay users were recruited based on the following inclusion criteria: both sexes, age 35-71 years, right- or left-handedness, and non-healthcare professionals. Exclusion criteria included a history of nasal surgery or known anatomic variation and being a healthcare professional. The participants were randomly assigned to one of two cohorts. The simulation cohort (n = 50; 38 female; mean age, 53.4 ± 2.6 years; 40 with prior nasal spray experience) administered the spray to a mannequin. The real-use cohort (n = 141; 63 female; mean age, 53.9 ± 18.7 years; 121 with prior experience) self-administered the spray under observation.

Task completion rates were evaluated against a prespecified 80% acceptance threshold. Including a margin of error of ±5% at a 95% confidence level and an 85% completion rate, a sample size of n = 195 was indicated. With statistical precision, 191 users were recruited.

Study Setting and Materials

**Figure 3:**
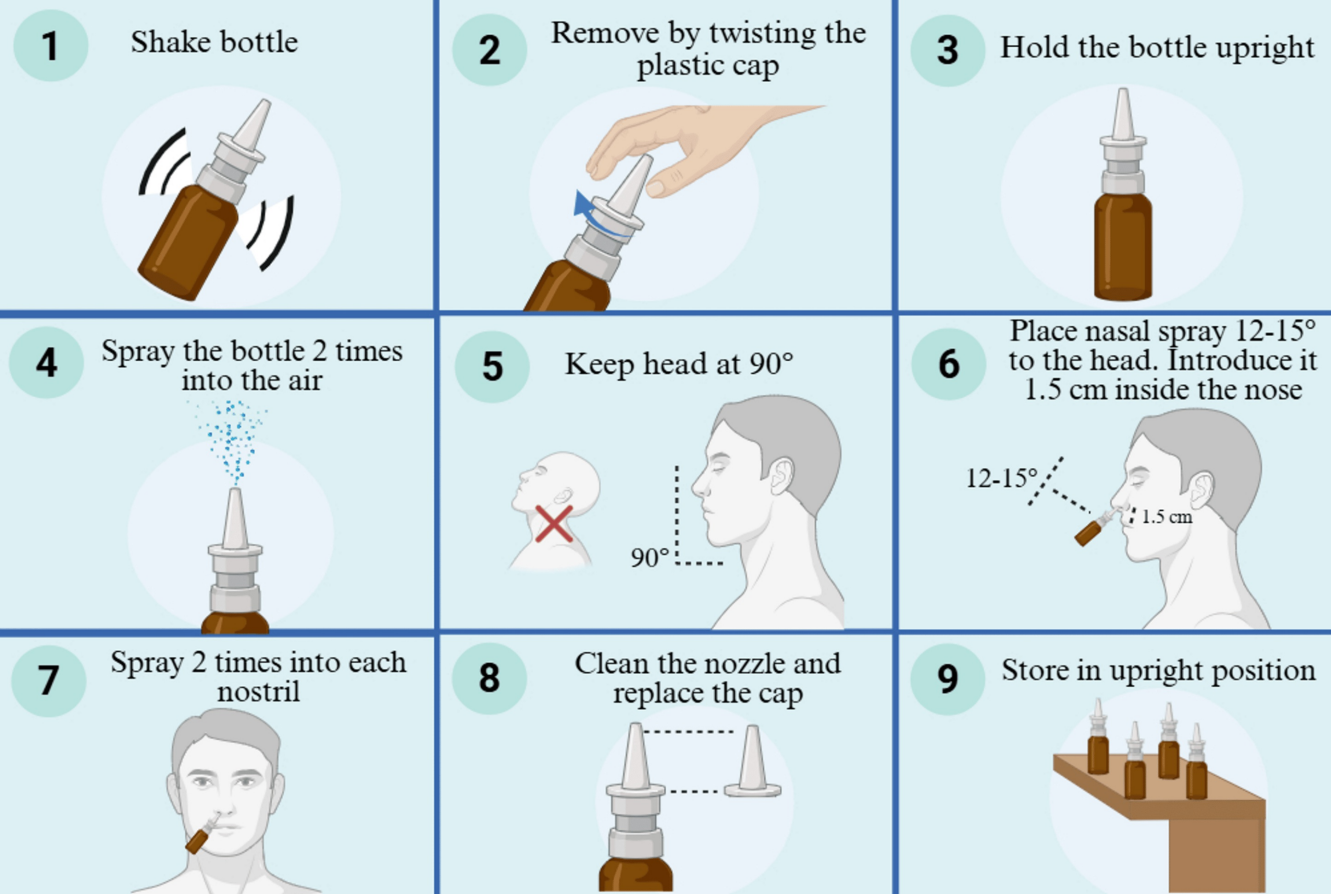
Visual aid provided to participants Visual aid provided to participants showing the recommended 12–15° nozzle angle relative to the head. The figure was manually created using BioRender.com (Science Suite Inc. d/b.a. BioRender, Toronto, Canada) under license.

Testing was conducted in nonclinical settings designed to approximate typical home-use conditions. Participants received the nasal pump sprayer (identical to that used in Part I), printed labeling with text instructions and visual diagrams (Figure 3), and a short instructional video demonstrating the optimized application technique. 

Task Protocol

The application procedure was deconstructed into nine steps across four stages: preparation (steps 1-4), device positioning (steps 5-6), delivery (step 7), and after use (steps 8-9) (Table 1). Steps 5, 6, and 7 were designated essential, as errors in these steps directly affect drug deposition at the target site. Observers recorded whether each participant completed each step correctly. A step-completion rate of 80% or higher was used as the predefined acceptance threshold, consistent with industry conventions for HF validation of combination products [19]. This completion rate indicates that the majority of users followed the crucial steps without error, meaning that nasopharyngeal drug penetration is not altered. Some level of user error was considered acceptable because an 80% completion rate translates to one in five users performing the crucial steps incorrectly.

**Table 1:** Application instructions for drug deposition

Stage	Step	Instruction
Preparation	1	Shake the spray bottle for 2–5 seconds
	2	Remove the plastic cap by twisting.
	3	Hold the bottle upright with two fingers on the pump shoulders and the thumb on the base.
	4	Prime by spraying twice into the air, away from the face
Device positioning	5	Keep head in neutral position (do not tilt forward or backward)
	6	Insert the nozzle at a 12–15° angle relative to the horizontal, as shown in the diagram.
Delivery	7	Deliver two sprays per nostril
After use	8	Wipe the nozzle with a tissue and replace the cap
	9	Store the bottle upright

Outcomes

The primary outcome was the step-completion rate for each of the nine steps in both cohorts. Secondary outcomes included the identification of the principal root causes of step failures and the global mean completion rate for each cohort.

Statistical analysis

For Part I, penetration per actuation (mL) was summarized as the median, interquartile range (IQR), and coefficient of variation (CV). Differences in median penetration between the 12-15° and 67.5° protocols were evaluated with Mann-Whitney U tests (right and left nostrils analyzed separately). Each trial was conducted as an independent actuation sequence, with the model being drained and repositioned between trials to maintain measurement independence. Given the exploratory nature of this study and the anatomically distinct nature of the right and left nostrils, which represent independent rather than multiple endpoints, no correction for multiple comparisons was applied. Significance was set at α = 0.05, two-sided.

Ethical considerations

The HF usability study was conducted as a product development human factors evaluation within the framework described in the FDA guidance on Human Factors Engineering for combination products [19,20]. The Code of Federal Regulations (CFR), Title 45, Public Welfare, Subtitle A, Department of Health and Human Services, Subchapter A, General Administration, Part 46, Protection of Human Subjects, Subpart A, Basic HHS Policy for Protection of Human Research Subjects, states that the study qualified for exemption from Institutional Review Board (IRB) review under 45 CFR §46.104(d)(3) because it met the criteria for interventions that are brief, harmless, not physically invasive, and not likely to have a significant adverse effect. Because participants prospectively agreed to participate in the study, all participants were verbally informed of the study purpose, the observational nature of data collection, and their right to withdraw at any time. No personal health data were collected.

## Results

Part I: In vitro nasopharyngeal penetration

The number of actuations required to collect 5.0 mL of posterior outflow and the corresponding median penetration per actuation for all four experimental conditions (two angles × two nostrils) are presented in Table 2. The results are from a single anatomic model and are not generalizable across diverse anatomies. At the 12-15° orientation, the right nostril was subjected to an average of 77.0 actuations (IQR, 3.7; CV, 4.8%), and the left nostril was subjected to an average of 88.8 actuations (IQR, 5.8; CV, 6.5%). At the 67.5° orientation, the corresponding values were 167.0 actuations (IQR, 8.2; CV, 4.9%) for the right nostril and 99.6 actuations (IQR, 6.0; CV, 6.0%) for the left nostril. 

**Table 2:** Comparative statistics of nasopharyngeal drug penetration volumes by nozzle angle orientation ^*^P-values: Mann-Whitney U test with exact computation (α = 0.05, two-sided). No multiple comparison correction applied, as nostrils represent independent anatomical units.

Nostril/angle	N	Median penetration (IQR) (mL)	P-value*	95% CI	Effect size (r)
Right nostril					
67.5	5	0.0296 (0.0287-0.0312)	Reference		
12-15	5	0.0649 (0.0625-0.0667)	0.008	0.0593-0.0705	0.84 (large)
Left nostril					
67.5	5	0.0505 (0.0500-0.0521)	Reference		
12-15	5	0.0581 (0.0532-0.0588)	0.028	0.0532-0.0588	0.70 (large)

**Figure 4:**
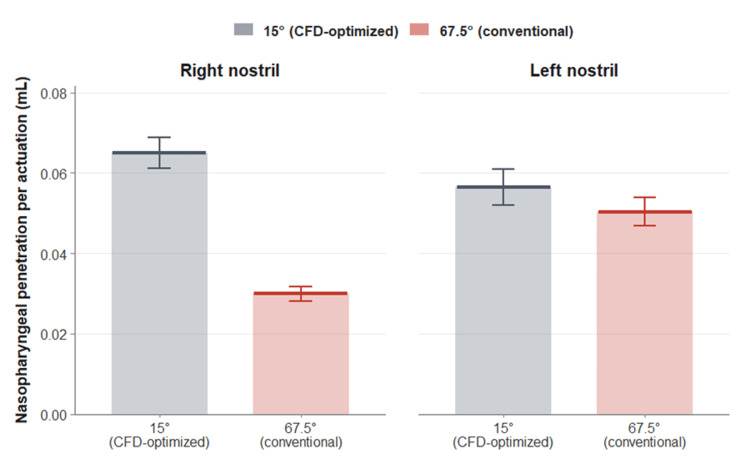
Confidence intervals between protocols in both nostrils Error bars indicate 95% confidence intervals (n = 5 trials per condition).

The 12-15° protocol delivered greater posterior nasal penetration than the 67.5° protocol in both nostrils (Figure 4). The effect was greater on the right side, where the median per-actuation outflow more than doubled, from 0.0296 mL (IQR, 0.0287-0.0312) at 67.5° to 0.0649 mL (IQR, 0.0625-0.0667) at 12-15°, a 119% relative increase (absolute difference, 0.0353 mL; U = 25.0; p = 0.008; r = 0.84). A similar effect was observed on the left, although of smaller magnitude: median outflow rose from 0.0505 mL (IQR, 0.0500-0.0521) to 0.0581 mL (IQR, 0.0532-0.0588), a 15% relative increase (absolute difference, 0.0076 mL; U = 23.5; p = 0.028; r = 0.70). Because each nostril represented an independent comparison, no adjustment for multiple comparisons was applied. The right nostril comparison (p = 0.008) remained significant even when a Bonferroni correction was applied (α = 0.025), whereas the left nostril comparison (p = 0.028) did not meet this threshold. 

The difference in magnitude between the right-nostril improvement (119%) and the left-nostril improvement (15.0%) was substantial. Review of the CT reconstruction revealed a visible septal deviation toward the left side in this donor anatomy, which may have reduced the effective cross-sectional area available for posterior plume penetration on the left.

Given the small sample size (n = 5 replicates per trial), penetration precision should be evaluated separately in each context. Robust findings were observed in the right nostril (obstructed path), with a large effect size (r = 0.84) and low variability (CV, 4.8%). In contrast, the left nostril improvement (r = 0.70; CV, 6.5%) approached the threshold. These findings suggest that results in obstructed anatomy may be less consistent; therefore, they should be considered preliminary, and further studies in diverse anatomies may be warranted.

Part II: Human factors usability

**Figure 5:**
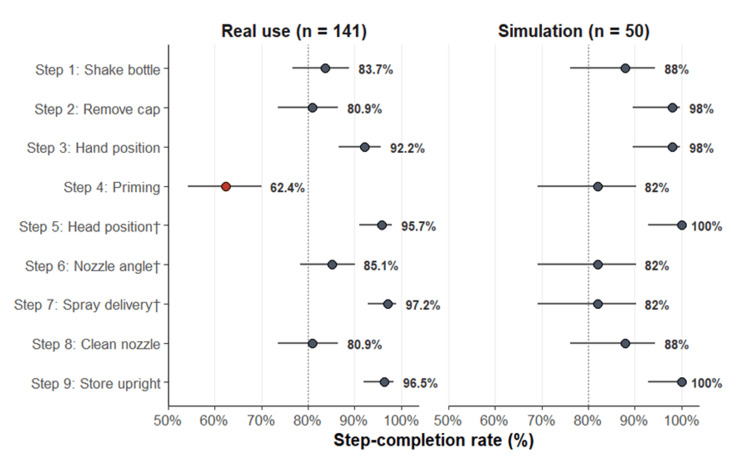
Step-completion rates Step-completion rates for the nine-step nasal spray protocol in the real-use and simulation cohorts. Filled circles indicate observed completion rates, horizontal whiskers denote 95% Wilson confidence intervals, and the dashed vertical line marks the prespecified 80% acceptance threshold. Essential steps are indicated by^ †^.

Step-completion rates for both cohorts are presented in Table 3 and Figure 5. As this is a descriptive rather than an inferential estimate, the reported IQRs represent medians across steps. In the simulation cohort, the global median completion rate across all nine steps was 90.8% (IQR, 8.2%). In the real-use cohort, it was 86.1% (IQR, 11.1%). All three essential steps (steps 5, 6, and 7) exceeded the 80% acceptance threshold in both cohorts: head position (step 5), 100% and 95.7% (95% CI, 91.0-98.0%); nozzle angle (step 6), 81.0% and 85.1% (95% CI, 78.3-90.0%); and spray delivery (step 7), 82.0% and 97.2% (95% CI, 93.0-98.9%), for the simulation and real-use cohorts, respectively. 

**Table 3:** Human factors task completion rates by cohort ^†^ Essential steps. ^* ^Below 80% acceptance threshold. CI, Wilson confidence interval.

Step	Description	Simulation (n = 50)	Real use (n = 141)	95% CI (real use)
1	Shake bottle	88.0%	83.6%	76.6–88.8%
2	Remove cap	98.0%	80.9%	73.6–86.5%
3	Hand position	98.0%	92.2%	86.6–95.6%
4	Priming	82.0%	62.4%*	54.2–70.0%
5^†^	Head position	100%	95.7%	91.0–98.0%
6^†^	Nozzle angle	82.0%	85.1%	78.3–90.0%
7^†^	Spray delivery	82.0%	97.2%	93.0–98.9%
8	Clean nozzle	88.0%	81.0%	73.7–86.6%
9	Store upright	100%	96.5%	92.0–98.5%
	Mean across steps	90.8 ± 8.2%	86.1 ± 11.1%	

The lowest-performing step overall was priming (step 4), which fell below the acceptance threshold in the real-use cohort at 62.4% (95% CI, 54.2-70.0%). Root-cause analysis attributed priming failures predominantly to users with prior nasal spray experience who felt sufficiently confident to skip this step. Nonadherence to step 1 (shaking the bottle) and step 2 (removing the cap) was attributed to participants who did not read the instructions (≥90% of failures in step 1) or who experienced difficulty with the twist-off cap, particularly among left-handed users (≥80% of failures in step 2).

## Discussion

This proof-of-concept and feasibility study provides initial experimental evidence for the hypothesis that a low-angle nozzle orientation (12-15°) increases nasopharyngeal spray penetration compared with the typically used orientation (67.5°). In the right nostril of a single CT-derived anatomic cast, the 12-15° protocol produced a 119% relative increase in median posterior outflow per actuation (p < 0.001). The improvement in the left nostril was more modest (15.0%; p = 0.028), likely reflecting the anatomic asymmetry inherent in the donor nasal anatomy, which exhibited visible septal deviation toward the left. These findings were obtained from a single anatomic cast; therefore, they should not be interpreted as clinical validation or evidence of therapeutic efficacy.

The meaningful improvement in the right nostril compared with the more modest improvement in the left nostril, 119% versus 15%, respectively, warrants consideration. The absolute differences between the two conditions (0.0353 mL and 0.0076 mL) represent the nasal spray dose; the bioavailability, clinical importance, and therapeutic effect should be established through pharmacokinetic studies. Although these results are consistent with prior CFD predictions by Akash et al. [8] and Basu et al. [10], who identified spray axis angle as the dominant modifiable parameter controlling posterior deposition.

From the human factors perspective, the optimized nine-step protocol proved feasible for lay users across both simulated and real-use conditions. All three essential steps, head position, nozzle angle, and spray delivery, exceeded the 80% completion threshold. The global completion rates (90.8% and 86.1%) compare favorably with published HF benchmarks for self-administered combination products [19]. However, priming emerged as a notable weak point, with only 62.4% of real-use participants performing this step. Root-cause analysis attributed the majority of failures to overconfidence among experienced users who skipped priming because of prior habits. This finding has direct design implications: labeling strategies that visually isolate and emphasize the priming step may improve adherence without adding instructional complexity.

The exploratory nature of this study must be emphasized when interpreting the findings. The use of a single anatomic cast limits the generalizability of the penetration estimates. Notably different improvements were obtained: 119% in the right nostril and 15% in the left nostril. Deposition outcomes were affected. The septal deviation in the donor anatomic cast explains the asymmetry in outcomes, but it also highlights a critical knowledge gap: there is no certainty that a 119% improvement can be achieved or that it will be altered in any way when applied to diverse anatomies, ages, or sexes. The results should be considered proof of concept in a unique anatomic cast rather than generalizable evidence; therefore, future studies should replicate these findings across anatomically diverse models.

Limitations

Several limitations constrain the generalizability of these findings and must be acknowledged. First, the in vitro tests were conducted using a single anatomic cast derived from a single adult male donor. The pronounced asymmetry between the nostrils demonstrates how individual nasal anatomy can modify deposition outcomes. It underscores the need for replication across anatomically diverse models (varying age, sex, ethnicity, and pathology). Second, the model lacked mucosal lining, mucociliary clearance, and simulated respiratory airflow; each of these factors influences post-deposition drug distribution in vivo and was not captured in the current design. Third, differences between the test solution and actual pharmaceutical formulations may affect atomization patterns and penetration dynamics.

All tests were performed using a proprietary nasal pump sprayer with specific characteristics, including atomization pressure, reduced discomfort, and droplet distribution, which may influence deposition patterns. The angle-dependent improvements obtained in this study may be device-specific, and further studies are required before adoption of this technique can be recommended, as different commercial nasal sprayers may differ in nozzle geometry, metering dose, or pump design.

In the HF component, the absence of objective angular measurement (such as goniometry or motion-capture video analysis) is a key limitation. Step 6 completion was scored by observation, and the degree to which participants actually achieved the 12-15° target was not quantified instrumentally; without instrumental verification, reproducibility is compromised. Sample size justification and power calculations were not specified a priori. Finally, the five-replicate design for the bench-top tests, while sufficient to detect the large right-nostril effect, limits precision for the smaller left-nostril difference. Additional limitations include: (a) observer bias, as evaluators were not blinded to the procedural steps under assessment; (b) participant recruitment was nonrandomized, which may represent selection bias, although no significant demographic differences (age, sex, or device experience) were identified; (c) uncertainty remains as to whether increased posterior outflow translates to improved therapeutic outcomes; (d) the trial order was not randomized, which could introduce bias; (e) lack of instrumental measurement of nozzle angles; (f) the lack of fluid property measurements restricts direct clinical extrapolation; and (g) a certain degree of uncertainty persists, as the increased nasopharyngeal penetration observed in vitro is not necessarily directly proportional to improved clinical outcomes in patients. However, no corrections for multiple comparisons were applied, as the right and left nostrils represent independent units. Therefore, individual p-values should be interpreted with caution because of the increased risk of type I errors. The right nostril result (p = 0.008) remained significant even when a Bonferroni correction was applied (α = 0.025), whereas the left nostril result (p = 0.028) would not meet this threshold, suggesting that this finding would benefit from replication.

Clinical implications and future directions

These data provide directional proof of concept that minor modifications to nozzle angle can meaningfully alter the regional distribution of a nasal spray plume. The clinical relevance of these findings is whether increased posterior outflow in a rigid cast translates to greater drug concentration at the nasopharyngeal epithelium in living subjects. This question requires pharmacokinetic or pharmacodynamic studies with mucosal sampling. Future investigations should include replication across multiple 3D-printed anatomies encompassing both sexes and diverse ethnicities, full rheological characterization of the test fluid, incorporation of simulated breathing and mucociliary transport, instrumented measurement of nozzle angle in the HF study (e.g., inertial sensors or video goniometry), stratified HF analyses by user experience, sex, and handedness, and prospective clinical trials assessing therapeutic outcomes of angle-optimized delivery in patients with viral upper respiratory infections.

## Conclusions

In a CT-derived anatomic nasal model, a 12-15° nozzle orientation produced greater nasopharyngeal penetration per actuation than the 67.5° orientation commonly specified in over-the-counter instructions, particularly in the unobstructed right nasal passage. Lay users followed a nine-step usage protocol for the optimized angle with acceptable fidelity for all essential tasks. These data provide preliminary in vitro and human factors data supporting angle-optimized intranasal delivery as an approach to improve nasopharyngeal drug deposition. Validation across anatomically diverse models and clinical populations is warranted before translation into clinical practice.
